# Income and Well-Being: Relative Income and Absolute Income Weaken Negative Emotion, but Only Relative Income Improves Positive Emotion

**DOI:** 10.3389/fpsyg.2016.02012

**Published:** 2016-12-21

**Authors:** Zonghuo Yu, Li Chen

**Affiliations:** ^1^Jiangxi Key Laboratory for Psychology and Cognitive Science and School of Psychology, Jiangxi Normal UniversityNanchang, China; ^2^Department of Applied Psychology, Wenzhou Medical UniversityWenzhou, China

**Keywords:** subjective well-being, negative emotion, positive emotion, multilevel analysis, relative income effect, absolute income effect

## Abstract

Whether relative income or absolute income could affect subjective well-being has been a bone of contention for years. Life satisfaction and the relative frequency of positive and negative emotions are parts of subjective well-being. According to the prospect theory, hedonic adaptation helps to explain why positive emotion is often so hard to be maintained, and negative emotion wouldn’t be easy to be eliminated. So we expect the relationship between income and positive emotion is different from that between income and negative emotion. Given that regional reference is the main comparison mechanism, effects of regional average income on regional average subjective well-being should be potentially zero if only relative income matters. Using multilevel analysis, we tested the hypotheses with a dataset of 30,144 individuals from 162 counties in China. The results suggested that household income at the individual level is associated with life satisfaction, happiness and negative emotions. On the contrary, at a county level, household income is only associated with negative emotion. In other words, happiness and life satisfaction was only associated with relative income, but negative emotion was associated with relative income and absolute income. Without social comparison, income doesn’t improve happiness, but it could weaken negative emotion. Therefore, it is possible for economic growth to weaken negative emotion without improving happiness. These findings also contribute to the current debate about the “Esterling paradox.”

## Introduction

It is a common belief that money makes people happier, but the relationship between income and subjective well-being is one of the most controversial issues in the research field of subjective well-being (e.g., Frey and Stutzer, 2002; Ferrer-i-Carbonell, 2005; Boyce et al., 2010; Easterlin et al., 2010; Easterlin, 2012). Many of the papers focused their attention on the mechanism underlying the relationship between income and subjective well-being. Some argued that both absolute income and relative income could affect subjective well-being, but others suggested that only relative income or rank of income played a role (e.g., Boyce et al., 2010). The “Easterlin Paradox” is one of the most famous theories about the relationship between income and happiness. In this theory, two factors, hedonic adaptation and social comparison, were regarded as the underlying psychological mechanisms for the relationship between individuals’ income and subjective well-being (Easterlin et al., 2010; Kahneman and Deaton, 2010; Easterlin, 2012). Consistent with what is predicted by the “Easterlin Paradox,” Deaton and Stone (2013) found relative income, but not absolute income, was significantly associated with happiness.

Although these studies have significantly advanced current knowledge concerning the relationship between individuals’ income and subjective well-being, there are still questions that require further clarification due to the methodological and theoretical limitation of the existing research on this topic. First, the relationship between income and subjective well-being among different regions/nations is vulnerable to be confounded by some factors such as culture, language, social institutions and so on (Seligman, 2004; Ye et al., 2015).

Second, individual’s subjective well-being could be evaluated from three aspects: emotional well-being, cognitive well-being and eudaimonic well-being (purpose). Emotional well-being consists of positive emotional well-being (such as happiness) and negative emotional well-being (such as depression, sadness, anxiety and so on) (OECD, 2013). However, negative emotional well-being was neglected in most studies of the relationship between income and subjective well-being (Kahneman and Deaton, 2010). According to the prospect theory, hedonic adaptation helps to explain why positive emotion is often so hard to be maintained, and negative emotion wouldn’t be easy to be eliminated (Tversky and Kahneman, 1992). Therefore, the relationship between income and positive emotion may be different from that between income and negative emotion.

Third, aggregating and disaggregating are both unsatisfactory for hierarchical data. Disaggregating all higher order variables to the individual-level may be contrary to the assumption of independence of observations. While aggregating individual characteristics over nations/regions may throw away all the within-group information, which often makes the relations between aggregated variables much stronger (Raudenbush and Bryk, 2002). Therefore, it’s necessary to apply new method to deal with hierarchical data.

### Current Research

An individual’s everyday life includes experiences of joy, stress, sadness, anger, and affection that make his life pleasant or unpleasant, so emotional well-being includes both positive emotion and negative emotion (Kahneman and Deaton, 2010). The objective of this paper is to test whether the relationship between income and positive emotional well-being is different from that between income and negative emotional well-being.

In the previous studies, negative emotions, such as depression, and positive emotions, such as happiness, were treated as the bipolar of a continuous dimension of emotion (e.g., Joseph and McCollam, 1993). However, a growing body of research on positive psychology has shown much evidence that positive emotion wasn’t simply the inverse of negative emotion (Seligman and Csikszentmihalyi, 2000). Meanwhile, a great deal of evidence also suggested that positive and negative emotions have independent effect on physical health (e.g., Kiecolt-Glaser et al., 2002; Fredrickson, 2003; Pressman and Cohen, 2005). For example, Kahneman and Deaton (2010) suggested that positive emotion and negative emotion were correlated weakly and were treated as different constructs. Furthermore, hedonic adaptation causes the pleasure of a raise to be transient (Kahneman and Deaton, 2010), so there is no permanent increase in happiness when a country’s income increases (Easterlin et al., 2010). Meanwhile, social comparison was regarded as the mechanism underlying the relationship between income and happiness (Deaton and Stone, 2013). Social comparison maybe also the mechanism underlying the relationship between income and negative emotion. For example, lower income may make people angry, anxious and so on. However, besides social comparison, absolute income may be another predictor of negative emotion. According to prospect theory, the pain of a loss was at least twice as potent as the pleasure generated by a gain (Tversky and Kahneman, 1992). Therefore, it may be difficult for people to adapt to negative emotion such as the pain of hunger, sickness or coldness which caused by low income. Based on the above analyses, it was hypothesized that relative income might significantly predict both negative emotion and positive emotion while absolute income could only significantly predict negative emotion.

In order to test the above-mentioned hypotheses, we adopted the method of multilevel data used in previous studies (Deaton and Stone, 2013): income at regional level data was used to measure the absolute income, while income at individual level data consisted of the absolute income and the relative income. An ecological correlation is almost certainly not equal to its corresponding individual correlation (Easterlin et al., 2010), so the method of multilevel analysis was adopted. Given that regional reference is the main comparison mechanism (Boyce et al., 2010) and social comparison is the underlying mechanism of the relationship between relative income and subjective well-being, if only relative income affects subjective well-being, the relationship between subjective well-being and income at individual-level will be significant, but that at regional level will be potentially zero (Deaton and Stone, 2013). On the contrary, if both relative income and absolute income are significantly associated with subjective well-being, the coefficient between subjective well-being and income will be significant at individual-level and regional level.

Therefore, according to the prospect theory, we expect that negative emotional well-being would be associated with income on the individual level and the regional level, but positive emotional well-being only would be associated with income on the individual level. Besides emotional well-being, we also tested the relationship between the cognitive aspect of subjective well-being and income, and we hypothesized that only relative income would affect their relationship. In other words, we expect that income would be significantly associate with life satisfaction at individual-level, but not at regional level.

In order to control confounding variables (e.g., language, culture and social institution), we used a representative dataset collected in China to examine the hypotheses. China is a fitting natural test case. First, China is more ethnically and politically unified than other regions in the world (Talhelm et al., 2014). Second, economic development of China is uneven. Levels of regional gross domestic product cover both ends of the spectrum from “third-world” to “first-world” (Yu and Wang, 2016). Data from a single pure ethnicity could control many irrelevant variables in cross-national comparisons, thus providing a more rigorous test of our hypotheses.

## Materials and Methods

### Data and Participants

The data used in this study was collected in 2010 by the Institute of Social Science Survey of Peking University, which has high quality and seemed as a survey to reveal true face of Chinese Society (Hvistendahl, 2010; Xie, 2012; Xie and Zhou, 2014), and we have received permission from the Institute of Social Science Survey of Peking University to use it. Participants were 32,905 individuals (16,952 females, 15,930 males; age ranging from16 to 110 years old, *M* = 45.55, *SD* = 16.39) from 162 counties in China. All participants received detailed information about CFPS and gave their consent to participate. 30,144 participants self-identified as ethnic Han Chinese, 2,761 as non-Han minorities. In order to exclude the effect of culture, we analyzed only Han Chinese (Talhelm et al., 2014).

### Measurement

#### Happiness

Happiness was measured with a single self-report five-point happiness item enquiring whether individuals were happy. Previous studies have found evidence pertaining to the validity of this specific single-item measure of happiness (Oswald and Wu, 2010).

#### Life Satisfaction

Life satisfaction measured with a single self-report five-point happiness item enquiring “How satisfied are you with your life as a whole these days?”

#### Negative Emotion

Negative Emotion was measured with six items by asking how often one felt depressed, agitated or upset, nervous, hopeless about future, and felt that everything was difficult or thought life was meaningless in the past month. Cronbach’s Alpha of these six items is 0.932.

#### Household Income per Capita

Household income per capita was the average income with the total income in the family divided by the family size. According to the basic fact of perception known as Weber’s Law, all of family income per capita (FIPC) are logarithmically transformed (e.g., Kahneman and Deaton, 2010).

#### Controlling Variables

Other demographic variables (e.g., age, gender, job status) were included as covariates at individual-level, and age, years of education and gender at county-level (e.g., Kahneman and Deaton, 2010; Deaton and Stone, 2013).

## Results

Up to 65.2% of respondents felt happy, and up to 50.1% of respondents had high life satisfaction. As shown in Supplementary Table 3 and 4, both two aspects of emotional well-being and cognitive well-being were significantly associated with income both at individual-level and at county-level. And respondents experienced low negative emotion (*M* = 2.74 for males, *M* = 3.33 for females, and total score was 30). For more details about correlation coefficients, and percentages of selected demographic, socioeconomic and health variables can be seen in the supplemental material (as shown in Supplementary Tables 1–4).

A multilevel random-coefficient model (Raudenbush and Bryk, 2002) was used to test the effect of household income per capita on emotional well-being and life satisfaction. Listwise deletion was used, and excluding missing data in some variables, the valid sample size was 23,029 in this model. The estimator of MLMV^1^ was used, which is robust to non-normality (Muthén and Muthén, 1998–2012). Some variables, such as marital status, educational status, age, job status, living in urban or rural, health status and gender, which could influence emotional well-being and life satisfaction, were controlled at the individual-level of the multilevel random coefficient model (e.g., Kahneman and Deaton, 2010). Other variables including gender, years of education and age were also controlled at county-level (e.g., Deaton and Stone, 2013). All non-dichotomous predictors were grand-mean-centered and entered as random slopes (e.g., Boyce et al., 2010).

The within-county variance accounted for 95.93% of variance of life satisfaction, 93.80% variance of happiness and 93.59% of variance of negative emotion. In this mutlilevel analysis, the statistical power for happiness, life satisfaction and depression at the county-level were respectively 0.885, 0.999 and 0.953. As shown in **Tables 1–3**, after other individual-level variables were controlled, logged household income per capita significantly predicted life satisfaction (β = 0.371, *p* < 0.01), happiness (β = 0.280, *p* < 0.01) and negative emotion (β = -0.596, *p* < 0.01) at the individual-level. However, as shown in **Tables 4–6**, at county-level, logged household income per capita significantly predicted negative emotion (*γ* = -1.838, *p* < 0.01), but didn’t significantly predict life satisfaction (*γ =* -0.142, *p =* 0.224) or happiness (γ = 0.023, *p* = 0.823).

**Table 1:** Mixed-effects linear model of individual level predictors of life satisfaction.

	Coefficient (*SE*)	95% CI	*P*-value
Urban	-0.063 (0.029)	[-0.119,-0.007]	0.027
Age	0.009 (0.001)	[0.007,0.011]	0.000
Gender	-0.077 (0.023)	[-0.123,-0.031]	0.001
Years of Educational	-0.002 (0.002)	[-0.006,0.002]	0.460
Height	0.002 (0.001)	[-0.001,0.004]	0.188
Smoke	-0.057 (0.019)	[-0.095,-0.019]	0.003
Have Child(ren)	-0.007 (0.044)	[-0.093,0.078]	0.867
Health	0.176 (0.010)	[0.157,0.196]	0.000
Family size	0.033 (0.005)	[0.023,0.043]	0.000
Household income per capita	0.371 (0.025)	[0.323,0.419]	0.000
Student	0.316 (0.037)	[0.244,0.388]	0.000
Retired	0.093 (0.029)	[0.036,0.151]	0.002
Keeping house	0.027 (0.031)	[-0.034,0.088]	0.390
Have job	-0.040 (0.023)	[-0.086,0.006]	0.088
Married	0.015 (0.047)	[-0.078,0.108]	0.750
Divorced or widowed	-0.161 (0.056)	[-0.272,-0.051]	0.004

*Gender (1 = Male,0 = Female); Marriage status (1 = unmarried; 2 = married; 3 = divorced or widowed).*

**Table 2:** Mixed-effects linear model of individual level predictors of happiness.

	Coefficient (*SE*)	95% CI	*P*-value
Urban	0.048 (0.029)	[-0.009,0.105]	0.097
Age	0.003 (0.001)	[0.001,0.004]	0.001
Gender	-0.068 (0.023)	[-0.114,-0.022]	0.004
Educational years	0.009 (0.002)	[0.005,0.012]	0.000
Height	0.002 (0.001)	[-0.001,0.004]	0.185
Smoke	-0.067 (0.018)	[-0.103,-0.032]	0.000
Have child(ren)	-0.136 (0.041)	[-0.216,-0.055]	0.001
Health	0.195 (0.009)	[0.177,0.213]	0.000
Family size	0.040 (0.005)	[0.030,0.050]	0.000
Household income per capita	0.280 (0.021)	[0.239,0.322]	0.000
Student	0.237 (0.054)	[0.131,0.344]	0.000
Retired	0.147 (0.034)	[0.082,0.213]	0.000
Keeping house	-0.010 (0.031)	[-0.072,0.051]	0.741
Have job	-0.035 (0.024)	[-0.083,0.012]	0.146
Married	0.189 (0.046)	[0.099,0.279]	0.000
Divorced or widowed	-0.058 (0.057)	[-0.171,0.054]	0.309

*Gender (1 = Male, 0 = Female); Marriage status (1 = unmarried; 2 = married; 3 = divorced or widowed).*

**Table 3:** Mixed-effects linear model of individual level predictors of negative emotion.

	Coefficient (*SE*)	95% CI	*P*-value
Urban	-0.082 (0.106)	[-0.289,0.126]	0.440
Age	-0.006 (0.003)	[-0.012,0.000]	0.034
Gender	-0.360 (0.068)	[-0.494,-0.226]	0.000
Educational years	-0.031 (0.007)	[-0.045,-0.016]	0.000
Height	-0.011 (0.005)	[-0.020,-0.002]	0.021
Smoke	0.234 (0.067)	[0.102,0.366]	0.001
Have child(ren)	-0.082 (0.127)	[-0.331,0.166]	0.515
Health	-1.268 (0.043)	[-1.352,-1.184]	0.000
Family size	-0.035 (0.020)	[-0.075,0.004]	0.079
Household income per capita	-0.596 (0.091)	[-0.773,-0.418]	0.000
Student	-0.152 (0.131)	[-0.409,0.105]	0.247
Retired	-0.740 (0.118)	[-0.972,-0.508]	0.000
Keeping house	-0.432 (0.115)	[-0.657,-0.207]	0.000
Have job	-0.188 (0.093)	[-0.370,-0.006]	0.043
Married	-0.533 (0.144)	[-0.816,-0.250]	0.000
Divorced or widowed	0.440 (0.170)	[0.106,0.774]	0.010

*Gender (1 = Male, 0 = Female); Marriage status (1 = unmarried; 2 = married; 3 = divorced or widowed).*

**Table 4:** Mixed-effects linear model of county level predictors of life satisfaction.

Independent variables	Coefficient (*SE*)	95% CI	*P*-value
Educational years	-0.011 (0.018)	[-0.046,.025]	0.554
Age	0.011 (0.010)	[-0.009,.031]	0.275
Gender	-1.416 (0.298)	[-2.001, 0.832]	0.000
Household income per capita	-0.142 (0.117)	[-0.372, 0.087]	0.224

**Table 5:** Mixed-effects linear model of county level predictors of happiness.

Independent variables	Coefficient (*SE*)	95% CI	*P*-value
Educational years	0.018 (0.016)	[-0.014, 0.049]	0.279
Age	-0.003 (0.009)	[-0.022, 0.015]	0.704
Gender	-1.874 (0.285)	[-2.431, 1.316]	0.000
Household income per capita	-0.023 (0.105)	[-0.228, 0.181]	0.823

**Table 6:** Mixed-effects linear model of county level predictors of negative emotion.

Independent variables	Coefficient (*SE*)	95% CI	*P*-value
Educational years	-0.127 (0.068)	[-0.261, 0.006]	0.062
Age	-0.069 (0.036)	[-0.140, 0.002]	0.058
Gender	-2.485 (1.234)	[-4.903,-0.066]	0.044
Household income per capita	-1.838 (0.466)	[-2.751, 0.924]	0.000

## Discussion

Two major contributions were provided in this paper to understanding the relationship between income and individual well-being. The first major finding is that the effect of income on emotional well-being at individual-level was different from that at county-level. In detail, income at individual-level was significantly associated with life satisfaction, happiness and negative emotion of individuals; however, income at county-level was only significantly associated with negative emotion, but not happiness or life satisfaction. Deaton and Stone (2013) suggested that effects of regional average income on regional average well-being should be potentially zero if only relative income matters. This study not only provided new evidence that only relative income was significantly associated with happiness and life satisfaction (Boyce et al., 2010), but that both absolute income and relative income were significantly associated with negative emotion. Based on the hedonic adaptation theory, although we argued that money cannot buy happiness unless social comparison works (Kahneman and Deaton, 2010), money could help people avoid hunger, coldness and so on, and the pain due to hunger or coldness is free from social comparison. The pains may be more potent than the pleasure (Tversky and Kahneman, 1992) and can’t be adapted. Therefore, the negative emotion was not only associated with relative income but also with absolute income. Meta-analysis showed that the correlation coefficient between money and happiness was often small, but effect sizes were larger in low-income developing economies (Howell and Howell, 2008). According to our research, we concluded that money could help people to be free from hunger and coldness, and relieve pains.

The second major finding is that the relation between money and subjective well-being can’t be totally explained by the theory “Easterlin Paradox.” Even though some previous studies showed that relative income but not absolute income was associated with happiness (e.g., Guan, 2010), the “Easterlin Paradox” theory still suggests income and happiness are positively associated among nations at a given time. However, the developmental levels among regions in China are very uneven and levels of regional GDPs cover both ends of the spectrum from “third-world” to “first-world” (Yu and Wang, 2016), but only 5∼7% of variance of subjective well-being is accounted by between-county variance, and most of variance of subjective well-being is accounted by within-county variance. Furthermore, different from the predictions made by the “Easterlin Paradox,” the result of this research shows that income and happiness aren’t significantly positively associated among counties.

Besides supplementing the theory of “Easterlin Paradox,” these new findings are helpful for policy making. Especially, even though “Easterlin Paradox” suggests economic growth in itself does not raise happiness, this study finds that there is significant correlational relationship between income and negative emotion. Therefore, we are confident that economic growth will weaken negative emotion, even though economic growth won’t improve happiness. Anyway, the study also stresses the implications of income distribution for subjective well-being, because relative income was associated with both happiness and negative emotion.

The sample used in this study minimized confounding factors such as cultures, ethnicity, languages and social institution, which made the conclusion in this study have a higher internal validity. Given that the construct of well-being may be different among cultures (Veronese and Pepe, 2014; Veronese et al., 2014), the research is helpful for the attempt of “de-centralizing” the idea of wellbeing from the western-centered approach. On the other hand, it should be acknowledged that this also limits generalizability. In Confucian culture, the pursuit of money is degraded to some degree, which may weaken the relationship between money and subjective well-being. So, whether the conclusion based on Chinese sample will be replicated in other cultures is still unknown.

## Ethics Statement

The data used in this article were supported by the project named China Family Panel Studies (CFPS), which was implemented by Institute of Social Science Survey, Peking University. All participants have given detailing information about CFPS and willing to take part in.

## Author Contributions

ZY designed the research and analyzed the data. ZY and LC wrote the manuscript and approved the final version of the manuscript for submission.

## Conflict of Interest Statement

The authors declare that the research was conducted in the absence of any commercial or financial relationships that could be construed as a potential conflict of interest.

## Footnotes

1 MLMV: maximum likelihood parameter estimates with standard errors and a mean- and variance-adjusted chi-square test statistic that is robust to non-normality.
