# Supplementary material for: Income and Well-Being: Relative Income and Absolute Income Weaken Negative Emotion, but Only Relative Income Improves Positive Emotion
Source: Front Psychol. 2016 Dec 21;7:2012. doi: 10.3389/fpsyg.2016.02012 (PMC5174105; doi:10.3389/fpsyg.2016.02012)
Supplement: Supplementary file 1 [file Table_1.DOCX]

## Supplemental material

| Table 1: Characteristics of the study population (Description of the continuous variables) | | | | | | | | | | | | | | | | | |
| --- | --- | --- | --- | --- | --- | --- | --- | --- | --- | --- | --- | --- | --- | --- | --- | --- | --- |
|  | | Age | | Years of education | | Height | | Happiness | Health | Life Satisfaction | | Negative Emotion | | Household income per capita | | Family size | |
| Mean | | 45.547 | | 6.484 | | 163.982 | | 3.872 | 1.79 | 3.508 | | 2.627 | | 3.806 | | 4.188 | |
| S.D. | | 16.39 | | 4.935 | | 8.017 | | 0.99 | 0.975 | 1.023 | | 3.039 | | 0.449 | | 1.789 | |
| Skewness | | 0.139 | | -0.01 | | -0.197 | | -0.646 | 1.35 | -0.367 | | 1.288 | | -0.547 | | 1.237 | |
| Kurtosis | | -0.644 | | -1.178 | | 1.472 | | -0.005 | 1.274 | -0.22 | | 1.166 | | 2.674 | | 4.905 | |
|  | |  | |  |  |  | |  |  |  | |  | |  | |  |  |

| Table 2: Characteristics of the study population(confined to Han nationality) | | | |
| --- | --- | --- | --- |
|  |  | Males(n=14657) | Females (n =15487) |
| Age(Mean, SD) |  | 45.44(16.327) | 46.05(16.479) |
| Type of neighborhood (n, %) | Urban | 6915(47.18%) | 7455(48.14%) |
|  | Rural | 7742(52.82%) | 8032(51.86%) |
| Job status (n, %) | Have job | 8039(56.34%) | 6390(41.63%) |
|  | Students | 850(5.96%) | 818(5.33%) |
|  | Retired | 469(3.29%) | 2265(14.75%) |
|  | Housework | 1465(10.27%) | 2045(13.32%) |
|  | No job | 3446(24.15%) | 3833(24.97%) |
| Marriage status (n, %) | Unmarried | 2314(15.80%) | 1658(10.71%) |
|  | Married | 11588(79.10%) | 12411(80.17%) |
|  | Divorced | 222(1.52%) | 166(1.07%) |
|  | Widowed | 525(3.58%) | 1245(8.04%) |

|  | uban | age | health | Years of education | gender | height | happy | Household income | Family size | smoke | child | Life satisfaction |
| --- | --- | --- | --- | --- | --- | --- | --- | --- | --- | --- | --- | --- |
| age | 0.006(0.321) |  |  |  |  |  |  |  |  |  |  |  |
| health | -0.069(0.000)** | 0.311(0.000)** |  |  |  |  |  |  |  |  |  |  |
| Years of education | 0.315(0.000)** | -0.433(0.000)** | -0.235(0.000)** |  |  |  |  |  |  |  |  |  |
| gender | -0.012(0.038)* | 0.021(0.000)** | -0.082(0.000)** | 0.147(0.000)** |  |  |  |  |  |  |  |  |
| height | 0.07(0.000)** | -0.171(0.000)** | -0.137(0.000)** | 0.27(0.000)** | 0.648(0.000)** |  |  |  |  |  |  |  |
| Happiness | 0.087(0.000)** | -0.020(0.001)** | -0.180(0.000)** | 0.116(0.000)** | -0.034(0.000)** | 0.037(0.000)** |  |  |  |  |  |  |
| Household income | 0.317(0.000)** | -0.030(0.000)** | -0.113(0.000)** | 0.333(0.000) | 0.008(0.181)** | 0.102(0.000)** | 0.150(0.000)** |  |  |  |  |  |
| Family size | -0.205(0.000)* | -0.143(0.000)** | -0.055(0.000)** | -0.087(0.000)** | -0.03(0.000) | -0.039(0.000)** | 0.006(0.292)** | -0.219(0.000) |  |  |  |  |
| smoke | -0.044(0.000)** | 0.074(0.000)** | -0.036(0.000)** | 0.007(0.209)** | 0.593(0.000) | 0.382(0.000)** | -0.041(0.000)** | -0.023(0.000)** | -0.012(0.045)** |  |  |  |
| child | -0.018(0.003)** | 0.565(0.000)** | 0.176(0.000)** | -0.29(0.000)** | -0.07(0.000)** | -0.146(0.000)** | -0.006(0.294) | -0.028(0.000)** | 0.046(0.000)** | 0.071(0.000)** |  |  |
| Life satisfaction | -0.025(0.000)** | 0.065(0.000)** | -0.132(0.000)** | -0.011(0.060) | -0.038(0.000)** | -0.006(0.317) | 0.490(0.000)** | 0.103(0.000)** | 0.027(0.000)** | -0.040(0.000)** | 0.023(0.000)** |  |
| Negative emotion | -0.112(0.000) | 0.013(0.031)* | 0.299(0.000)** | -0.126(0.000)** | -0.073(0.000)** | -0.08(0.000)** | -0.254(0.000)** | -0.158(0.000)** | 0.023(0.000)** | -0.02(0.001)** | -0.023(0.000)** | -0.230(0.000)** |

| Table 3: Correlation coefficients among three aspects of subjective well-being(at individual-level )   \|  \| uban \| age \| health \| Years of education \| gender \| height \| happy \| Household income \| Family size \| smoke \| child \| Life satisfaction \| \| --- \| --- \| --- \| --- \| --- \| --- \| --- \| --- \| --- \| --- \| --- \| --- \| --- \| \| age \| 0.006(0.321) \|  \|  \|  \|  \|  \|  \|  \|  \|  \|  \|  \| \| health \| -0.069(0.000)** \| 0.311(0.000)** \|  \|  \|  \|  \|  \|  \|  \|  \|  \|  \| \| Years of education \| 0.315(0.000)** \| -0.433(0.000)** \| -0.235(0.000)** \|  \|  \|  \|  \|  \|  \|  \|  \|  \| \| gender \| -0.012(0.038)* \| 0.021(0.000)** \| -0.082(0.000)** \| 0.147(0.000)** \|  \|  \|  \|  \|  \|  \|  \|  \| \| height \| 0.07(0.000)** \| -0.171(0.000)** \| -0.137(0.000)** \| 0.27(0.000)** \| 0.648(0.000)** \|  \|  \|  \|  \|  \|  \|  \| \| Happiness \| 0.087(0.000)** \| -0.020(0.001)** \| -0.180(0.000)** \| 0.116(0.000)** \| -0.034(0.000)** \| 0.037(0.000)** \|  \|  \|  \|  \|  \|  \| \| Household income \| 0.317(0.000)** \| -0.030(0.000)** \| -0.113(0.000)** \| 0.333(0.000) \| 0.008(0.181)** \| 0.102(0.000)** \| 0.150(0.000)** \|  \|  \|  \|  \|  \| \| Family size \| -0.205(0.000)* \| -0.143(0.000)** \| -0.055(0.000)** \| -0.087(0.000)** \| -0.03(0.000) \| -0.039(0.000)** \| 0.006(0.292)** \| -0.219(0.000) \|  \|  \|  \|  \| \| smoke \| -0.044(0.000)** \| 0.074(0.000)** \| -0.036(0.000)** \| 0.007(0.209)** \| 0.593(0.000) \| 0.382(0.000)** \| -0.041(0.000)** \| -0.023(0.000)** \| -0.012(0.045)** \|  \|  \|  \| \| child \| -0.018(0.003)** \| 0.565(0.000)** \| 0.176(0.000)** \| -0.29(0.000)** \| -0.07(0.000)** \| -0.146(0.000)** \| -0.006(0.294) \| -0.028(0.000)** \| 0.046(0.000)** \| 0.071(0.000)** \|  \|  \| \| Life satisfaction \| -0.025(0.000)** \| 0.065(0.000)** \| -0.132(0.000)** \| -0.011(0.060) \| -0.038(0.000)** \| -0.006(0.317) \| 0.490(0.000)** \| 0.103(0.000)** \| 0.027(0.000)** \| -0.040(0.000)** \| 0.023(0.000)** \|  \| \| Negative emotion \| -0.112(0.000) \| 0.013(0.031)* \| 0.299(0.000)** \| -0.126(0.000)** \| -0.073(0.000)** \| -0.08(0.000)** \| -0.254(0.000)** \| -0.158(0.000)** \| 0.023(0.000)** \| -0.02(0.001)** \| -0.023(0.000)** \| -0.230(0.000)** \|   * *p*<.05. ** *p*<.01.    Table 4: Correlation coefficients among three aspects of subjective well-being (at county-level) | | | |  |  |
| --- | --- | --- | --- | --- | --- | --- | --- | --- | --- | --- | --- | --- | --- | --- | --- | --- | --- | --- | --- | --- | --- | --- | --- | --- | --- | --- | --- | --- | --- | --- | --- | --- | --- | --- | --- | --- | --- | --- | --- | --- | --- | --- | --- | --- | --- | --- | --- | --- | --- | --- | --- | --- | --- | --- | --- | --- | --- | --- | --- | --- | --- | --- | --- | --- | --- | --- | --- | --- | --- | --- | --- | --- | --- | --- | --- | --- | --- | --- | --- | --- | --- | --- | --- | --- | --- | --- | --- | --- | --- | --- | --- | --- | --- | --- | --- | --- | --- | --- | --- | --- | --- | --- | --- | --- | --- | --- | --- | --- | --- | --- | --- | --- | --- | --- | --- | --- | --- | --- | --- | --- | --- | --- | --- | --- | --- | --- | --- | --- | --- | --- | --- | --- | --- | --- | --- | --- | --- | --- | --- | --- | --- | --- | --- | --- | --- | --- | --- | --- | --- | --- | --- | --- | --- | --- | --- | --- | --- | --- | --- | --- | --- | --- | --- | --- | --- | --- | --- | --- | --- | --- | --- | --- | --- | --- |
|  | Life satisfaction | Happiness | | |  |
| Life satisfaction |  |  |  |  |  |
| Happiness | 0.024(0.005)^***^ |  |  |  |  |
| Negative emotion | -0.060(0.018)^**^ | -0.063(0.019)^**^ |  |  |  |
| *Note*. ** *p*<.005. *** *p*<.001. | | | | | |
